# Management of the General Process of Parenteral Nutrition Using mHealth Technologies: Evaluation and Validation Study

**DOI:** 10.2196/mhealth.9896

**Published:** 2018-04-03

**Authors:** Mercedes Cervera Peris, Víctor Manuel Alonso Rorís, Juan Manuel Santos Gago, Luis Álvarez Sabucedo, Carmina Wanden-Berghe, Javier Sanz-Valero

**Affiliations:** ^1^ Pharmacy Service University Hospital Son Espases Palma Spain; ^2^ Department of Telematics Engineering Telecommunication Engineering School University of Vigo Vigo Spain; ^3^ Health and Biomedical Research Institute of Alicante University General Hospital of Alicante Alicante Spain; ^4^ Department of Public Health and History of Science School of Medicine Miguel Hernandez University Alicante Spain

**Keywords:** parenteral nutrition, mobile apps, quality control, validation software

## Abstract

**Background:**

Any system applied to the control of parenteral nutrition (PN) ought to prove that the process meets the established requirements and include a repository of records to allow evaluation of the information about PN processes at any time.

**Objective:**

The goal of the research was to evaluate the mobile health (mHealth) app and validate its effectiveness in monitoring the management of the PN process.

**Methods:**

We studied the evaluation and validation of the general process of PN using an mHealth app. The units of analysis were the PN bags prepared and administered at the Son Espases University Hospital, Palma, Spain, from June 1 to September 6, 2016. For the evaluation of the app, we used the Poststudy System Usability Questionnaire and subsequent analysis with the Cronbach alpha coefficient. Validation was performed by checking the compliance of control for all operations on each of the stages (validation and transcription of the prescription, preparation, conservation, and administration) and by monitoring the operative control points and critical control points.

**Results:**

The results obtained from 387 bags were analyzed, with 30 interruptions of administration. The fulfillment of stages was 100%, including noncritical nonconformities in the storage control. The average deviation in the weight of the bags was less than 5%, and the infusion time did not present deviations greater than 1 hour.

**Conclusions:**

The developed app successfully passed the evaluation and validation tests and was implemented to perform the monitoring procedures for the overall PN process. A new mobile solution to manage the quality and traceability of sensitive medicines such as blood-derivative drugs and hazardous drugs derived from this project is currently being deployed.

## Introduction

The use of information and communication technologies (ICTs) in the health field opens a long list of possibilities aimed at achieving benefits at many levels in the health environment. A wide spectrum of research trends emerges. The use of ICTs plays a paramount role in the development of innovative clinical nutrition projects, especially in collaborative environments that go far beyond Web apps. A new area emerges, eHealth, in which great advances, new benefits, and possibilities arise when applying ICT to the health care domain, especially with the use of mobile apps, mHealth.

Systems supported by telematic apps enable registries that allow linking and evaluation of information on parenteral nutrition (PN) processes at any time [1]. The activity of verification of a system developed for PN control allows determining if it meets the requirements and conditions imposed in the design phase [2] and allows defining the tasks the telematic app is expected to perform. Verification should confirm that the system correctly implements its specification. Control operations must validate that hazards are monitored and ensure that the system works as planned. Likewise, the developed system must document the registries of the controls established to demonstrate that the entire PN process is under control and corrective actions have been taken in the event of any deviation from the critical points [3].

The good practices guide for the preparation of medicines in hospital pharmacy services establishes that all procedures related to the preparation of medicines, including PN, should be approved and reviewed. This guide establishes the need to evaluate and verify all procedures related to the preparation of medicines under the premise of “applying the principles of risk management for quality and quality by design” [4]. The European Commission, in its guide on principles and guidelines for good manufacturing practices for medicines [5], pointed out the need for qualification and validation of facilities, equipment, services, processes, protocols, etc. The quality by design implied systematized and continuous validation of the processes in order to improve them based on the scientific evidence, always taking into account quality, efficiency, and safety [4].

The Spanish Society of Hospital Pharmacy (Sociedad Española de Farmacia Hospitalaria), in its standards of practice for specialized nutritional support established by its Nutrition Working Group [6], recommended “defining the tools of the management process aimed at achieving an efficient nutritional support, which allow obtaining the best results with a reasonable cost.” One of the standards that helped achieve this goal was developed by identifying the problems that affected quality, evaluation of the processes, and verification of the results.

The objective of this study was to evaluate the mHealth app and validate its effectiveness in monitoring the management of the PN process.

## Methods

### Design of the Study

A follow-up study was planned to evaluate a previously developed mHealth app and validate that it covers the real needs of the general process of PN.

### Unit of Analysis

The units were the PN bags prepared by the pharmacy service of the Son Espases University Hospital (HUSE) of Palma, Spain.

### Identification of the Parenteral Nutrition Bags

For the management of the stages of validation and transcription of the prescription, HospiWin 2000 version 8 (Baxter SL) software was used. This program provided the label that was attached to each PN bag. In addition, the entities to be monitored (PN bags) were identified with a unique code (a QR label was attached).

### Study Period

The tracking of the PN bags was carried out from June 1 to September 6, 2016.

### Determination of the Parenteral Nutrition Process

The flow diagram, including all the stages of traceability and management of the PN, was generated and is published elsewhere [7]. Using this flowchart as the starting point, a dashboard to monitor the involved stages was created selecting the critical control points (CCPs).

### mHealth App

**Figure 1:**
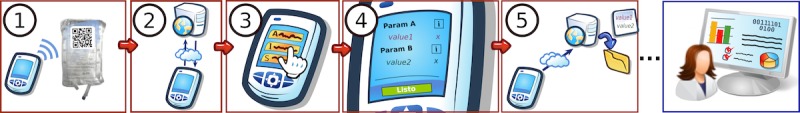
General behavior of the software platform.

Using a methodology based on hazard analysis and taking advantage of semantic technologies, a holistic service platform for traceability and process control was designed. Figure 1 summarizes the operation of the platform.

Upon the reading of a QR tag attached to an element to be monitored (in this case a PN bag), a data exchange occurs between the mobile app and the analysis server (steps 1 and 2). This server accesses its semantic knowledge base to infer the potential control operations associated with the PN bag. Using this data and other contextual information (such as the user type, location, or time), a personalized list of ranked operations is sent to the mobile app (steps 2 and 3). The user selects the most convenient operation (step 3), probably the first one on the list, and the app generates a form to capture the values required for the task under consideration (step 4). At this point, the human user completes the form and captures photos when required, and the mobile app acquires any other required pieces of data, such as GPS location or time. These data are sent to the server to update the knowledge base (step 5). Finally, analysts and auditors can use a Web dashboard to visualize the data and trace the operations. A more detailed description of the platform is published elsewhere [8,9].

The verification of this app was previously published [10]. The app was deployed in an environment of home hospitalization to test its capacity to geoposition the stages and, in particular, the medicine administration stage [3].

### Invocation of the mHealth App

Each one of the requests performed by this app was linked to a certain control operation in a particular stage. In case an operation was invoked more than once, by PN bag, the system only validated the first request, storing the remaining ones as “null requests.”

### Evaluation of the mHealth App

An assessment of the utility and ease of use was performed. For this purpose, the Poststudy System Usability Questionnaire (PSSUQ) [11] was used. This questionnaire was designed to collect the perception of the participant in this type of study. It consists of 19 items, uses a 7-point Likert scale, and allows estimating the perceived value of usability over 4 dimensions: general satisfaction (OVERALL), utility of the app (SYSUSE), quality of the information (INFOQUAL), and quality of the interface (INTERQUAL). Based on the collected data, reliability of the responses was determined. The survey was generated in electronic format and sent to 50 professionals at HUSE with no advanced knowledge in ICT. They could answer anonymously.

### Validation of the Parenteral Nutrition System

Validation was performed by checking the compliance of all the operations in each of the stages and corroborating that they were adequately monitored, particularly the operative control points (OCPs) and, more importantly, the CCPs. In order to validate the process, the results obtained from 387 PN bags at HUSE were analyzed.

In the preparation stage for the gravimetric control, according to HUSE protocol for PN bags with a volume greater than 100 mL, the real weight should not exceed a margin of ±5% of its theoretical weight.

For control of the storage temperature of the PN bags according to the procedure for the preservation of thermolabile medications at HUSE [12], the operational limits were established at 2ºC (lower limit) and 8ºC (upper limit) and the critical limits at 0.1ºC and 12ºC.

In the administration stage, in the absence of preestablished limits for time and speed infusion, recommendations for each specific nutrition were observed according to the patient's clinical and social situation and the device used for its administration.

### Variables to Study

The stages of the process were prescription, preparation, preservation, and administration. All control operations at each stage of the process were recorded in a dichotomous way (yes/no), according to whether they were finished or not.

The operations characterized as CCPs were gravimetric control, temperature of the refrigerator, and volume and time of infusion (administration). These operations were recorded in a continuous and quantitative manner.

The operations characterized as OCPs were microbiological control (which is recorded a posteriori), final check of the PN (including the checking of physical parameters such as turbidity, precipitate, strange color, etc), and existence of the filter in the infusion pump (according to the PN type). These operations were recorded by means of dichotomous observations (yes/no). In all cases, it was possible to capture an image to prove compliance with a certain operation.

### Data Analysis

The parameters monitored through the mobile device (phone or tablet) were sent to the Web server. This server processed these data and stored them in the knowledge base in the form of facts. The information model used was designed using Semantic Web–based techniques that allowed representation of concepts and relationships and offered advanced query services and analysis capabilities [9].

To access the information managed by the system, the Web service offered a dashboard accessible through a common Web browser. In this dashboard, analysts and auditors were able to study and evaluate the history of records generated in any phase of the process, filter them based on various aspects of the context (date, responsible user, etc), download the information in a standardized format (comma-separated values), and visualize usage statistics through easily interpretable graphics.

A descriptive analysis was performed on the control operations assigned to each stage. The quantitative variables were characterized by their sample mean, standard deviation, median, mode, maximum, minimum, and interquartile interval (IQI). To avoid problems caused by outliers in the mean, the robust average was also calculated. The qualitative variables were characterized by their absolute and relative frequencies. Figures and tables were used to represent the most relevant qualitative variables.

For analysis of the internal consistency of the answers obtained from the PSSUQ (study of the reliability of the measurement scale), a Cronbach alpha coefficient was used. To evaluate the significance in the difference of means for independent samples, a Student *t* test was used. The significance level used in all hypothesis tests was alpha≤.05.

Quality control of the data was performed through double tables and active search for errors. When errors were found, they were corrected by consulting the original source. SPSS Statistics version 22.0 (IBM Corp) was used for the analysis.

### Ethical Requirements

This work is part of the PI13/00464 project and was approved by the project evaluation body of the Miguel Hernández University with registration number 2013.95.E.OEP.

## Results

During the implementation of the mHealth system for the traceability and management of PN at HUSE, 50 professionals took part and a total of 387 PN bags were managed.

### Evaluation of the mHealth App

The usability test was performed at HUSE, with a total of 21 hospital professionals collaborating (42% of the staff was invited to participate).

**Table 1:** Results according to the dimensions of the Poststudy System Usability Questionnaire [11].

Usability dimensions	Description	Mean (SD)	Median^a^	Cronbach alpha^b^
OVERALL	Overall satisfaction	5.1 (1.9)	6	.98
SYSUSE	System usefulness	5.2 (1.8)	6	.94
INFOQUAL	Information quality	5.8 (1.9)	6	.96
INTERQUAL	Interface quality	4.8 (2.0)	5	.98

^a^Maximum value: 7.

^b^Maximum value: 1.

**Table 2:** Control operations for each stage of the parenteral nutrition management system at Son Espases University Hospital.

Stage	System control operation	Adult (n=353), n	Pediatric (n=34), n
Validation of the prescription	Validate prescription	353	34
Transcription of the prescription	Transcribe prescription	353	34
Preparation	Initial check of trays/materials Control of PN^a^ preparation Final check of trays/materials Gravimetric control (CCP^b^) Adequacy of physical parameters (OCP^c^) Final check of PN (OCP) Microbiological record of PN (OCP)	353^d^	34^d^
Conservation	Temperature control of the refrigerator (CCP)	353	34
Interruption/suspension of administration	Cause of interruption/suspension of the administration^e^	26	4
Administration	Start time Infusion time (CCP) Volume to be administered (CCP) Identification of the pump Existence of the filter (OCP)	327	30

^a^PN: parenteral nutrition.

^b^CCP: critical control point.

^c^OCP: operative control point.

^d^The records regarding the microbiological analysis of the PN and, where appropriate, the counter sample were generated a posteriori once the results were known.

^e^Suspension: complications due to underlying disease or complications related to PN (mechanical, infectious, metabolic). Interruption: improvement (oral/enteral), transfer to another center or exitus.

The overall result of the responses to the questionnaire presented the following values: mean 96.0 (SD 6.6), median 102, minimum 29, maximum 133, and IQI 72.5 to 118.5 (maximum score 133 = 19 items ×7 maximum score). The statistics obtained according to the different usability dimensions and study of the reliability of the measurement scale using the Cronbach alpha coefficient can be seen in Table 1.

### Validation of the Parenteral Nutrition System

In the validation study, 387 PN bags were managed; 91.2% (353/387) of the bags were from adult patients and 8.8% (34/387) from pediatric patients, with a total of 3860 control operations (see Table 2). A total of 7.8% (30/387) of the bags were interrupted in administration, 6.7% (26/387) in adults and 1.0% (4/387) in pediatrics: 2.1% (8/387) changed to oral/enteral nutrition, 2.3% (9/387) due to the underlying disease, and 3.4% (13/387) due to complications related to PN (mechanical, infectious, or metabolic issues). These data proved that all operations could be registered in the digital repository.

### Dichotomic Controls of the Operative Control Point

All operations related to OCP were recorded and audited in the knowledge base, including the type of operation, date, and person who carried out the operation. Compliance with these controls was 100%.

### Quantitative Controls of the Critical Control Points (Gravimetric, Infusion Time, and Volume to Be Administered)

The statistics obtained from the control of the operations labeled as CCPs for PN bags intended for adults and children can be checked in Table 3. Compliance with CCP control was 100%.

**Figure 2:**
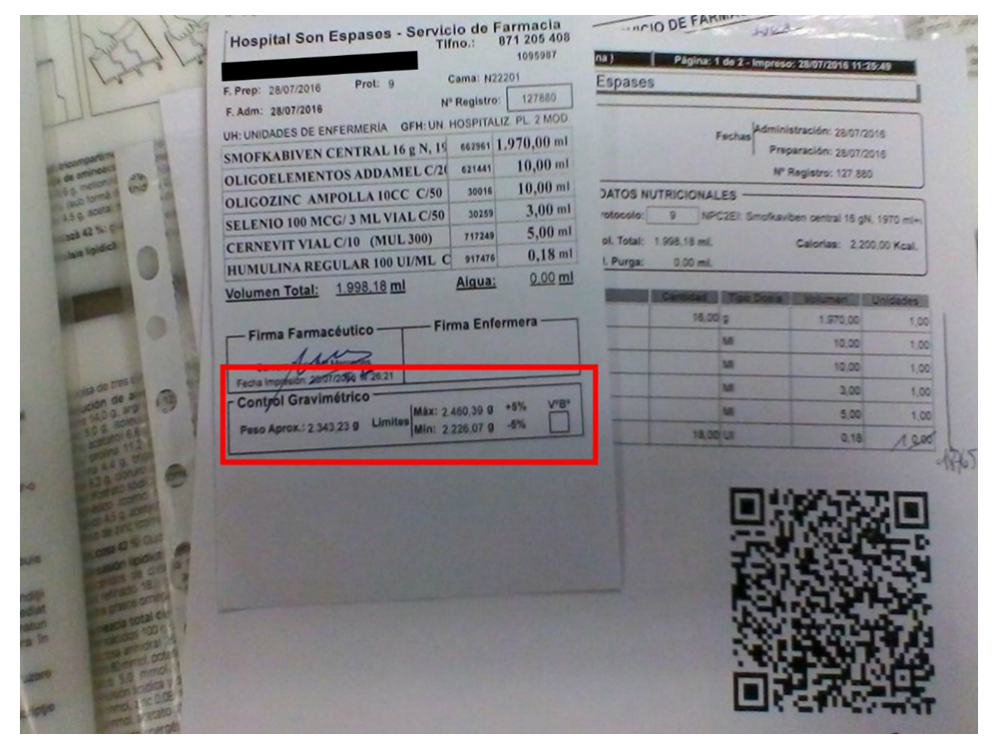
Label on a bag of parenteral nutrition, including data from the gravimetric control.

As shown on Table 3, the gravimetric controls allowed verification that deviation of weight of the bags was 0.5% in adult PN and 4.9% in pediatric PN (both measurements were within the range of allowed deviation: ±5% of the theoretical weight). In addition, the gravimetric control of each bag and its registration by image allowed verification that all were within the expected weight (see Figure 2).

In the administration stage, it was found that for infusion time (measured in hours), the deviation from the mean was 0.3 hours for adults and 0 hours for children (see Table 3).

**Table 3:** Statistics regarding the control operations identified as critical control points in the management system and the traceability control of parenteral nutrition.

Patient, stage, and control operation			Mean (SD)	Robust mean	Median	Max	Min
**Adult**							
	**Preparation**						
		Gravimetric control (g)	2182.0 (11.3)	2195.0	2285	2885	1730
	**Administration**						
		Infusion time (h)	22.2 (0.3)	21.7	24	48	11
		Volume to be administered (mL)	1868.8 (10.8)	1886.5	1998	2028.4	1200
**Pediatric**							
	**Preparation**						
		Gravimetric control (g)	937.9 (46.8)	936.0	955	1270	640
	**Administration**						
		Infusion time (h)	24.0 (0)	24.0	24	24	24
		Volume to be administered (mL)	736.7 (58.4)	703.5	700	2028	300

### Temperature Monitoring in the Refrigeration Chamber

**Figure 3:**
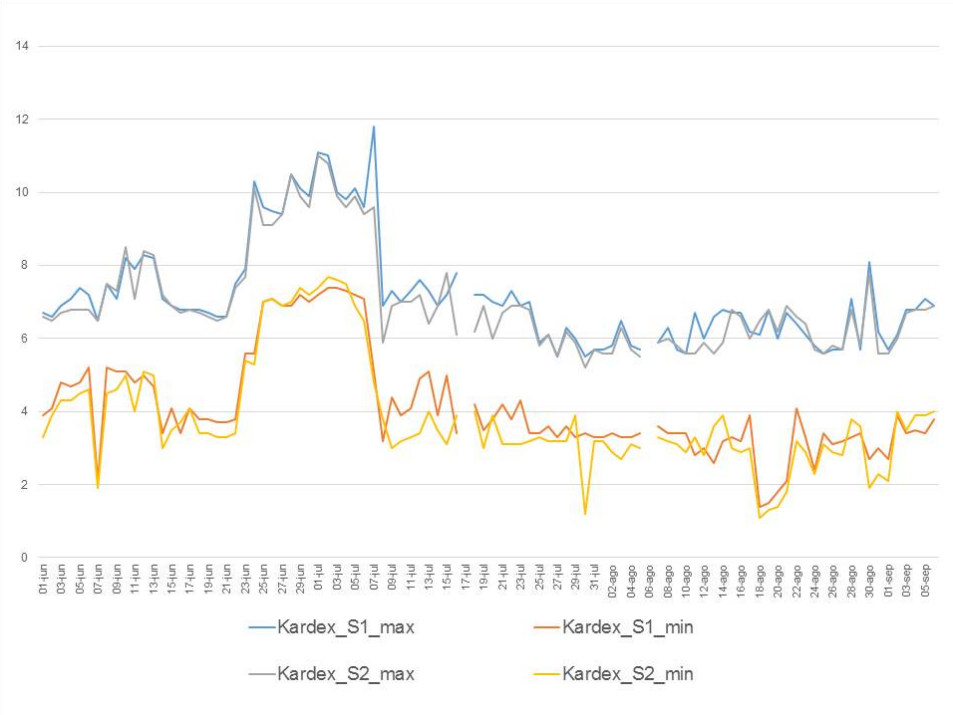
Maximum and minimum temperatures measured by the 2 probes in the refrigeration chamber from June 1, 2016 to September 6, 2016.

The results obtained in the period under consideration from daily recording of the temperature by 2 probes in the refrigeration room (Kardex Remstar, BioCold Environmental Inc) can be seen in Figure 3.

A nonconformity was detected from June 24 to July 7 that did not influence the validity of the PN bags since although the operative limit was exceeded (8ºC), the critical limit was not reached (12ºC). Likewise, there was a 10-day period when the temperature was lower than 2ºC, although in no case was it below 1.1ºC, so the temperature never fell below the critical limit (0.1ºC). In both situations, measures described in the established protocol had to be adopted [12]. On July 17 and August 6, the temperature in the refrigeration room was not registered (nonconformity). All nonconformities were recorded in the digital repository.

The statistics for data collected from the temperature sensors in the refrigeration room were:

- Maximum temperature (in ºC):
  - Probe 1 (P1): mean 7.2 (SD 0.1) (CI 95% 6.9-7.5); robust average 7.1; median 6.9; maximum 11.8; minimum 5.5; and IQI (6.1-7.5)
  - Probe 2 (P2): mean 7.0 (SD 0.1) (CI 95% 6.7-7.3); robust average 6.9; median 6.7; maximum 11.0; minimum 5.2; and IQI (5.9-7.2)

Comparison of means between P1 and P2 was *t*_190_=1.0; *P*=.31. There were no statistical differences in the measure of the maximum temperature between the 2 probes.

- Minimum temperature (in ºC):
  - Probe 1 (P1): mean 4.1 (SD 0.1) (CI 95% 3.8-4.4); robust average 4.1; median 3.8; maximum 7.4; minimum 1.4; and IQI (3.3-4.8)
  - Probe 2 (P2): mean 3.8 (SD 0.2) (CI 95% 3.5-4.2); robust average 3.7; median 3.4; maximum 7.7; minimum 1.1, and IQI (3.0-4.6)

Comparison of means between P1 and P2 was *t*_190_=1.3; *P*=.19. There were no statistical differences in the measure of the minimum temperature between the 2 probes.

### Management Controls

**Figure 4:**
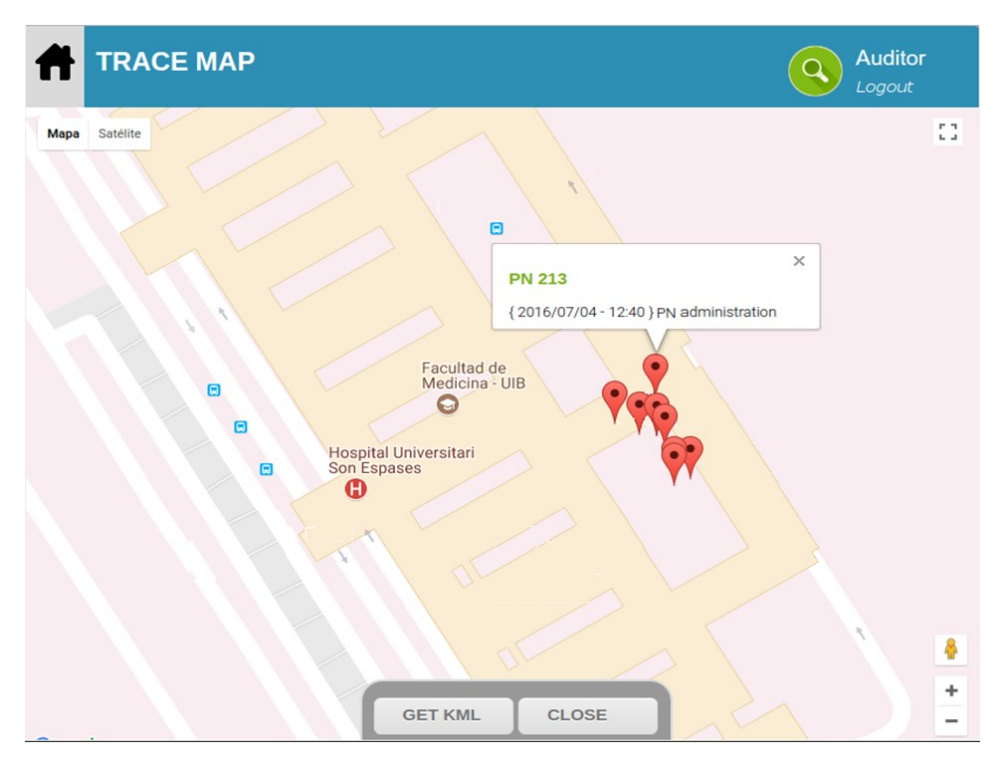
Collected data about the parenteral nutrition administration, including location, date, and time of administration. PN: parenteral nutrition.

The geolocation function provided by the app made it possible to know the location, day, and hour of administration of every PN bag (see Figure 4).

## Discussion

### Principal Findings

The evaluation and validation performed verified the ability of the mHealth app to register all designed processes and record nonconformities that occurred. Therefore, it was found that holistic monitoring of the traceability and management system of the PN could be implemented.

The results of the evaluation of the mHealth app through a survey revealed that, in general, users assessed its usability positively. The 4 dimensions measured (general satisfaction, app utility, information quality, and interface quality), on average, tended to the highest values ​​of the Likert scale used. This means that the professionals considered the mHealth app useful in their work, and they found it easy to manage even without technological experience. The calculated Cronbach alpha (with values ​​close to 1) allowed us to be sure about the consistency of the evaluation test (ie, the absence of errors in the measurements made). This result showed that the positive perception of the app is common to all the users who took part in the process.

The validation of the system allowed us to observe the monitoring and registration of all stages of the PN system. As already stated in the scientific literature, compliance with protocols minimizes unjustified clinical variability, which directly affects the reduction of risks associated with PN [13] and even facilitates the improvement of clinical skills [14]. It should be pointed out that the possibility of controlling and registering the trays with the initial and final preparation products and the postprocessing verification of the PN bags added an additional guarantee to the process. The repository of the registers in each one of the stages records the deviations (nonconformities) and, consequently, allows us to apply the necessary corrective actions to improve the whole PN process.

The characterization and quantification of CCPs was essential to know to what extent the process designed met the quality standards (scope or degree of compliance with the standards) [15]. The empirical controls complied with the recommendations of the standardized work procedures established at HUSE and the Spanish consensus on the preparation of parenteral nutrient mixtures [16]. According to Siebert et al [17], although research in this area is limited, it is important to develop and evaluate mobile apps that reduce the rate of medication errors.

All bags prepared were of volumes greater than 100 mL, so the margin of error of actual weight compared to theoretical weight should not exceed 5%. These results are in line with previous studies on gravimetric control in the PN process [17-20], which in any case met the established standards. The registration by image of each one of the PN bags allowed verification that the real weight of each was within the range of the expected weight.

In the administration stage, characterized mainly by the speed or rate of infusion of PN (volume to be infused per time), there are no specific regulations. Usually, only recommendations based on the clinical situation of the patient and device used for administration are established.

A margin of ±1 hour of the scheduled infusion time is a sensible recommendation, and the reasons for larger deviations should be evaluated [21]. In this study, the mean deviation of the infusion time met the established margins.

Other data on administration, such as location, date, and time, made it possible to collect very important data on control of the processes and holistic traceability of the PN. A large number of errors associated with the use of PN occur at the administration stage. Therefore, every stage of the process must be properly followed and managed. It is appropriate to establish procedures, especially in home care, that provide alerts about unwanted deviations [22]. The software app allowed us to ensure the validity of the conservation temperature and avoid variations on the compatibility and stability of the PN, which contributes to patient safety, one of the relevant dimensions in the quality of health care [23]. This control allowed us to detect the exceeding of the operating limit that occurred and lack of compliance during 2 days of the temperature record of the refrigeration room. These nonconformities were collected and studied from the history of the digital repository.

The use of a geographic information system made it possible to be certain about the disposition of the PN and get additional information about administration of the PN. In home care, the app would allow us to be aware of the actual arrival date of the PN at the patient’s home [3]. The benefits from the use of Web 2.0 tools, such as Mashups, as well as Web 3.0 or Semantic Web technologies have been demonstrated previously [24] and can be very useful to track the adherence to nutritional treatment.

But mobile apps not only help the adherence to treatment; the traceability also contributes to minimizing security issues related to custody of the data and controlling the integrity of the medicine itself [25].

In any case, in a context of progressive penetration of mobile apps for the management and traceability of medicines, it is desirable for health professionals to contribute by increasing health literacy (necessary to adequately recognize risks) and proper decision-making to take advantage of the opportunities inherent in Medicine 2.0 [26].

Furthermore, as has been demonstrated in previous projects, the use of mobile apps designed to guide the medication-related processes significantly reduces, compared to conventional preparation methods, the medicine preparation time, administration time, and rate of medication errors [27].

From the point of view of the authors, it is clear that the use of mobile apps for the management of medication, including parenteral nutrition, is a hot research topic with a bright future [3,26,28].

### Limitations

To control the volume being administered, it is difficult to establish a margin. The PN has a particular volume. Therefore, the potential hazard is linked to infusions done in less time than established, which would generally be due to a failure in the infusion pump [29]. To avoid errors in the rate of infusion, MacKay et al [30] proposed the establishment of minimum and maximum limits and double verification against the electronic prescription data. These advices were implemented by the app developed.

In its recommendations [21], the American Society for Parenteral and Enteral Nutrition emphasizes safety measures that avoid potential issues related to the infusion pump. Whenever possible, the pumps must be standardized and there must be operating protocols (including rules about silencing the alarm) to reduce errors due to programming issues. Avoiding these errors and capturing the data can support quality improvement programs. Beside all these issues, it also must be noted that ad hoc training on the procedures concerning the infusion pump for patients and caregivers, especially in home care, must be provided [3,31]. All of these recommendations were taken into account when deploying the CCPs of the proposed PN process.

### Conclusion

From the results obtained, we have concluded that the developed app successfully passed the evaluation and validation tests and, more importantly, properly completed the procedures for monitoring the overall PN process. In addition, the repository of records allowed us to see the deviations at all times. Therefore, a system for the management and traceability of PN that quantifies the main control points at a low cost has been successfully implemented and verified at HUSE in Palma, Spain.

A new mobile solution to manage the quality and traceability of sensitive medicines such as blood-derivative drugs and hazardous drugs derived from this project is currently being deployed.

## Abbreviations

CCP: critical control point
HUSE: Son Espases University Hospital
ICT: information and communication technology
IQI: interquartile interval
OCP: operative control point
PN: parenteral nutrition
PSSUQ: Poststudy System Usability Questionnaire
